# Structural and Functional Characterisation of TesA - A Novel Lysophospholipase A from *Pseudomonas aeruginosa*

**DOI:** 10.1371/journal.pone.0069125

**Published:** 2013-07-18

**Authors:** Filip Kovačić, Joachim Granzin, Susanne Wilhelm, Biserka Kojić-Prodić, Renu Batra-Safferling, Karl-Erich Jaeger

**Affiliations:** 1 Institut für Molekulare Enzymtechnologie, Heinrich-Heine Universität Düsseldorf, Forschungszentrum Jülich, Jülich, Germany; 2 Institute of Complex Systems (ICS-6), Forschungszentrum Jülich, Jülich, Germany; 3 Department of Physical Chemistry, Rudjer Bošković Institute, Zagreb, Croatia; SUNY Upstate Medical University, United States of America

## Abstract

TesA from *Pseudomonas aeruginosa* belongs to the GDSL hydrolase family of serine esterases and lipases that possess a broad substrate- and regiospecificity. It shows high sequence homology to TAP, a multifunctional enzyme from *Escherichia coli* exhibiting thioesterase, lysophospholipase A, protease and arylesterase activities. Recently, we demonstrated high arylesterase activity for TesA, but only minor thioesterase and no protease activity. Here, we present a comparative analysis of TesA and TAP at the structural, biochemical and physiological levels. The crystal structure of TesA was determined at 1.9 Å and structural differences were identified, providing a possible explanation for the differences in substrate specificities. The comparison of TesA with other GDSL-hydrolase structures revealed that the flexibility of active-site loops significantly affects their substrate specificity. This assumption was tested using a rational approach: we have engineered the putative coenzyme A thioester binding site of *E. coli* TAP into TesA of *P. aeruginosa* by introducing mutations D17S and L162R. This TesA variant showed increased thioesterase activity comparable to that of TAP. TesA is the first lysophospholipase A described for the opportunistic human pathogen *P. aeruginosa*. The enzyme is localized in the periplasm and may exert important functions in the homeostasis of phospholipids or detoxification of lysophospholipids.

## Introduction

The GDSL-family of lipolytic enzymes [1] is significantly different from other lipolytic enzyme families belonging to the so-called α/β-hydrolases [2,3]. In contrast to the canonical α/β-hydrolase fold, enzymes grouped in the GDSL family contain the active-site serine residue in a conserved Gly–Asp–Ser–Leu (GDSL) motif instead of the usually occurring pentapeptide Gly-Xaa-Ser-Xaa-Gly (GXSXG) [4,5]. The GDSL enzymes have catalytic residues, namely Ser, Gly, Asn and His, located in consensus sequences designated as blocks I, II, III, and V, respectively (Figure S1). The latter observation led to the proposal of the new name SGNH-hydrolase family that is now used as a synonym to the GDSL-hydrolase family. While the catalytic serine enclosed in the GXSXG-motif of α/β-hydrolases is located approximately in the middle of the amino acid sequence [3], the GDSL-motif is positioned close to the N-terminal end [2]. An additional distinguishing feature is the distance of catalytic triad residues histidine and aspartate in the sequence, which are separated by two amino acids in the GDSL-hydrolases, and by 50 or more amino acids in α/β-hydrolases. Differences in the consensus sequences around the catalytic triads of GDSL- and GXSXG-hydrolases are reflected in considerable differences of their three dimensional structures [2,6]. For example, the nucleophilic elbow, a characteristic structural feature strongly conserved among α/β-hydrolases and important for catalysis, is absent in GDSL-hydrolases [3]. Furthermore, GDSL-hydrolases are characterised by a three-layered α/β/α-fold with a conserved core structure consisting of five β-strands and at least four α-helices [2,6]. The presence of additional secondary structural elements inserted in the canonical α/β/α-fold and differences in the loops building the substrate-binding site point to evolutionary differentiation within a common fold [7]. A structural evolution of GDSL-hydrolases is supported by a broad phylogenetic distribution of these enzymes; they are found in bacteria, archaea, mammals, plants, fungi, and even viruses [2,8].

To date, only few structural studies of GDSL hydrolases have been reported [6,9–11]. Despite less than 20% sequence identity, their overall fold proved to be conserved. One of the unique characteristics reported for GDSL enzymes is their broad substrate specificity and multi-functionality [12]. In a previous report, we examined the substrate promiscuity in several GDSL-enzymes including the lipase SrLip from *Streptomyces* *rimosus* (Q93MW7), the two lipolytic enzymes EstA (from *Pseudomonas aeruginosa*, O33407), EstP (from *Pseudomonas* *putida* , Q88QS0) and the esterase TesA from *P. aeruginosa* (Q9HZY8) studied here [12]. Activity assays with 34 different substrates typical for esterases, thioesterases, lipases, phospholipases, Tweenases and proteases revealed SrLip as promiscuous enzyme, whereas TesA, EstA and EstP were shown to possess mainly esterase activity with different affinities and catalytic efficacies towards *p*-nitrophenyl butyrate. Interestingly, TesA from *P. aeruginosa* shows high sequence homology to the well-characterised multifunctional enzyme TAP from *Escherichia coli* that exhibits thioesterase, lysophospholipase A, protease and arylesterase activities [13]. TesA shows high arylesterase activity, but only minor thioesterase and no protease activity [12]. These obvious differences between TesA and TAP prompted us to carry out a detailed comparison of these two enzymes on biochemical, structural and physiological levels.

Here we present the cellular localisation, functional characterisation and three dimensional structure of TesA from *P. aeruginosa*. Beside the esterase EstA and the acetylcholinesterase ChoE, TesA is the third known GDSL hydrolase from *P. aeruginosa*. ChoE from *P. aeruginosa* is a one domain protein which shares only 13% sequence identity with TesA [14], its three-dimensional structure is not available. The outer membrane esterase EstA is an autotransporter enzyme [15] that comprises two domains, an N-terminal catalytic esterase (GDSL) domain exposed on the cell surface and a C-terminal β-barrel domain forming a channel in the outer membrane [15]. Despite a low sequence similarity to the EstA catalytic domain, the X-ray structure of TesA revealed that they share the same fold, as well as a similar composition of the substrate binding site. Furthermore, we have performed a systematic biochemical and structural analysis of *P. aeruginosa* TesA and *E. coli* TAP to address the issue of substrate promiscuity among these enzymes. Superposition of TesA and TAP revealed a structural diversity in the thioester binding site, where TesA shows significantly lower thioesterase activity. Using site-directed mutagenesis, we introduced point mutations substituting TesA residues Asp17 and Leu162 for the structurally equivalent residues of TAP, namely Ser18 and Arg160. The resulting TesA variants with the engineered thioester binding site showed a 2.2-fold increase in thioesterase activity, confirming our hypothesis that the differences in enzyme activities can be assigned to minor structural differences in the putative thioester binding site. Finally, the periplasmic localisation of TesA in the host organism *P. aeruginosa* PA01 and its high catalytic efficiency towards natural lysophospholipid substrates suggest an important role in lysophospholipid homeostasis. We therefore discuss the putative physiological role of TesA in *P. aeruginosa*, an opportunistic pathogen with significant medical, environmental and biotechnological relevance [16,17].

## Materials and Methods

### Protein localisation

The *tesA* gene was cloned in the pET22b vector using 5´-TAACATATGCGTGCATTGCTG-3´ and 5´-TAAGAGCTCTAACTCGAGAAGCAGCGGTTTCAG-3´ oligonucleotide pair as described earlier for pET22b-TesAH6, where TesAH6 refers to the presence of a 6×His tag in the C-terminus [12]. The subcloning from pET22b-TesA was then performed into pBBR1mcs-3 plasmid using *Xba*I and *Sac*I restriction sites yielding pBBR1mcs3-TesA (Table S1).

Both the broad host range plasmid pBBR1mcs3 that served as an empty vector control and pBBR1mcs3-TesA were conjugationally transferred from *E. coli* S17.1 into the *P. aeruginosa* PA01 host using biparental spot mating. The strains and plasmids used in this study are described in Table S1. Overnight cultures of *E. coli* S17.1 carrying plasmids were inoculated at an optical density OD_580nm_ of 0.05 and grown at 37^°^C in LB medium supplemented with 10 µg/ml tetracycline until they reached logarithmic phase, at an OD_580nm_ 0.5-0.8. The *E. coli* S17.1 in log-phase and the overnight culture of *P. aeruginosa* PA01 were then mixed in a volume ratio of 2:1 and spotted to the LB agar plate followed by incubation overnight at 37°C. *P. aeruginosa* transconjugants were selected on LB-agar plates supplemented with 25 µg/ml irgasan and 100 µg/ml tetracycline.

*P. aeruginosa* cultures, expressing *tesA* (or the empty vector control) were harvested in early stationary phase (OD_580nm_^~^ 1) by centrifugation at 3000 *g* for 10 min at 4°C, and fractionated using a modified protocol of Witholt et al. [18]. Residual cells were removed from the supernatant by filtration using the pore size of 0.2 µm. To release periplasmic proteins, cells were resuspended in Tris-HCl buffer (100 mM, pH 8) supplemented with 10% (w/v) sucrose to yield cell suspension with OD_580nm_ of 10 in 1 ml. An equal volume of Tris-HCl buffer (100 mM, pH 8) supplemented with 10% (w/v) sucrose and 5 mM EDTA was then added. Subsequently, the cell suspension was incubated with lysosyme (1500 U) for 30 min at room temperature with gentle shaking. The periplasmic proteins released into the supernatant were separated from the spheroblasts by centrifugation at 10000 *g* for 20 min at 4°C. The spheroblasts were disintegrated by sonication (Sonifier W250; Branson) and centrifuged at 3000 *g* for 10 min at 4°C to remove the inclusion bodies and the cell debris. The total cell membrane fraction, including the outer and inner membranes, was collected by ultracentrifugation at 180000 *g* (TLA-55 rotor) for 1 h at 4°C.

### SDS-PAGE and zymographic analysis

Proteins were analysed by polyacrylamide gel electrophoresis under denaturation conditions (SDS-PAGE) on 14% (w/v) gels as described by Laemmli (1970) [19]. Esterase activity in SDS-PAGE gel was detected by zymography using 4-methylumbelliferyl butyrate (MUB) as substrate. Before activity detection, TesA was refolded by incubating the gel two times for 30 min in Tris-HCl buffer (100 mM pH 8) supplemented with 25% (v/v) propan-2-ol at 4°C. Subsequently, the gel was incubated for 10 min in 5 mM MUB dissolved in Tris-HCl (100 mM, pH 8) containing 25% (v/v) propan-2-ol and fluorescence was detected with an Eagle Eye II video imaging system (Stratagene) [20].

### Enzyme activity assays and kinetic studies

Esterase and thioesterase activities towards *p*-nitrophenyl esters and acyl-coenzyme A thioesters were determined as described previously [12]. Lysophospholipase A activities towards 1-hexyl-glycerophosphocholine (C6-GPC), 1-lauryl-glycerophosphocholine (C12-GPC) and 1-palmitoyl-glycerophosphocholine (C16-GPC) were determined over a range of substrate concentrations from 0.5 mM to 5 mM. The reaction mixtures of 100 µl containing lysoPL substrate, 1.5 mM NaN_3_, 0.25% (v/v) Triton X-100, 20 mM Tris-HCl pH 7.2 and 1 µg of TesA were incubated at 37°C for 30 min. Lysophospholipase A activities towards 1-stearoyl-glycerophosphocholine (C18-GPC) and 1-oleoyl-glycerophosphocholine (C18: 1-GPC) were determined at substrate concentrations of 0.67 mM as described for C6-GPC. Prior to incubation, lysoPLs were vortexed for 15 min at 37°C and then exposed to ultrasonication thrice for 20 seconds [21]. C6-GPC, C12-GPC and C16-GPC were purchased from Avanti Polar Lipids (Alabaster, AL, USA) and C18-GPC and C18: 1-GPC from Sigma Aldrich (St. Louis, MO, USA). Released fatty acids were quantified spectrophotometrically in 96-well plates using NEFA-HR(2) Kit (Waco Chemicals) [22]. The fatty acid amount was calculated from the calibration curve using oleic acid at concentrations ranging from 0.1 to 3.5 mM. Kinetic parameters, *K*_m_ and *k*_cat_, were calculated from three independent experiments, where the data were fitted to the Michaelis-Menten equation using a non-linear regression method.

### Protein expression, purification, site-directed mutagenesis and crystallisation

Recombinant TesAH6 containing 207 residues including an N-terminal 21-residue signal peptide and C-terminal His_6_-tag was expressed in *E. coli* BL21(DE3) and purified as described previously [12]. Amino acid substitutions in *tesAH6* gene were performed in two steps by the Quik-change PCR method using *Pfu* DNA polymerase (Invitrogen). In the first step, pET22b-TesAH6 [12] plasmid was used as a template with complementary mutagenic oligonucleotide pair (mutated codons are underlined and nucleotides of wild type gene are indicated in subscript) 5´-GCCGCTTTGGGACTGA ^G^ G ^A^ TACCAGCCAGGGCTG-3´/5´-CAGCCCTGGCTGGTACT ^C^ C ^T^ AGTCCCAAAGCGGC-3´ for mutation of Asp17 into Ser. The resulting mutated plasmid pET22b-TesAH6_D17S was then used as a template with oligonucleotide pair 5´-CATCCGGCGC G ^T^ CGCCGCCCAG-3´/5´-GTAGGCCGCG C ^A^ GCGGCGGGTC-3´ for the mutation of Leu162 into Arg. The presence of desired nucleotide substitutions was confirmed by DNA sequencing. The TesA D17S/L162R variant was expressed and purified as described for TesAH6 wild type.

For crystallisation purpose, TesAH6 eluted from the Ni-NTA column with buffer containing 250 mM imidazole was exchanged with Tris-HCl buffer (50 mM, pH 8.0) and concentrated to 10 mg/ml using an ultrafiltration device with a membrane of 5 kDa pore size. The protein concentration was determined by the method of Bradford [23]. Crystals were grown at 19°C using the sitting-drop vapour diffusion method by mixing 1 µL of 10 mg/mL protein and 1 µL of reservoir solution (20% (w/v) PEG 3350, 50mM sodium-citrate buffer, pH 4.5). Typically, crystals appeared within 5 days and were cryo-protected using 10% (w/v) glycerol before storage in liquid nitrogen for data collection.

### X-ray diffraction data collection, three dimensional structure determination and refinement

X-ray diffraction dataset was collected at 100K. Native data were recorded at beamline ID14-1 of the ESRF (Grenoble, France) on a ADSC Quantum Q210 CCD detector system using a wavelength of 0.9334 Å. Data processing including reflections up to 1.9 Å resolution was carried out using MOSFLM [24] and SCALA, which are part of the CCP4 software suite [25].

The crystals obtained for TesA belonged to space group C2. The structure was determined by molecular replacement with PHASER [26] with a single native dataset. The search model was created with MODELLER [27] using the crystal structure of the EstA esterase (PDB code: 3HP4). Crystals were found to contain two protein molecules per asymmetric unit, corresponding to a Matthews coefficient of 2.29 Å^3^/Da and a solvent content of 46.3%. Model improvement was achieved by automated rebuilding cycles and additional positional and isotropic temperature factor refinement (PHENIX package). For manual rebuilding the program COOT [28] was used.

Graphics were generated with PyMol [29], MOLSCRIPT [30] and RASTER3D [31] using secondary structure assignments as given by the DSSP method [32]. The atomic coordinates and structure factors (code 4JGG) have been deposited in the Protein Data Bank (www.rcsb.org) [33].

## Results and Discussion

### TesA is a lysophospholipase A localised in the cell periplasm

**Figure 1:**
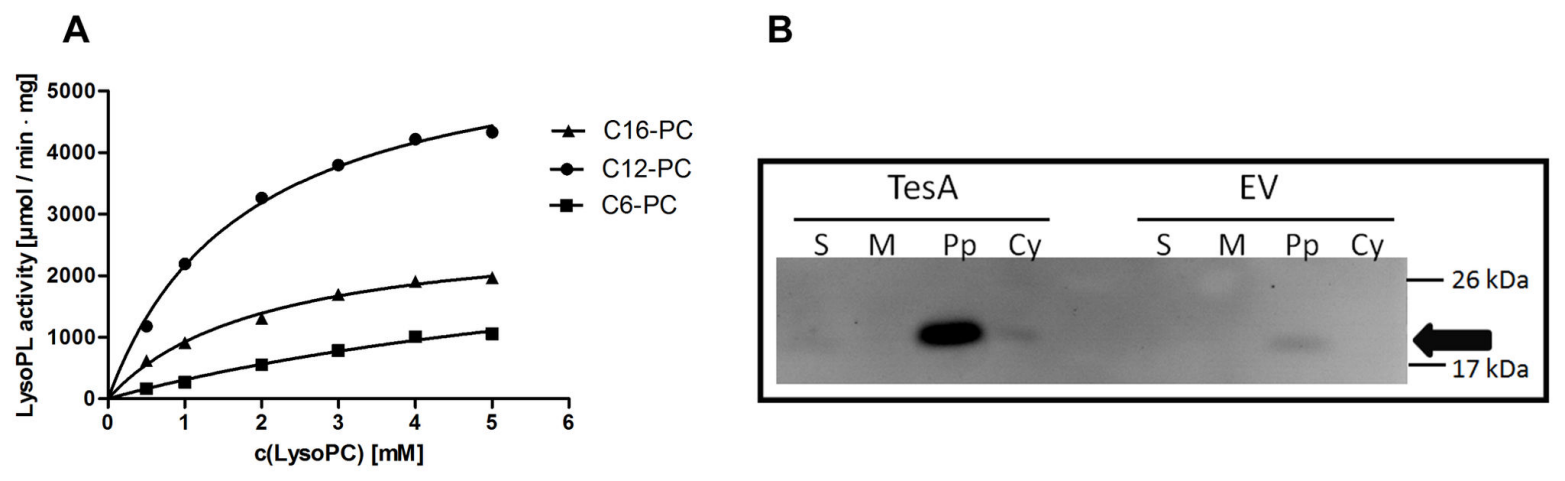
Enzyme kinetics and localization of TesA. (A) Enzyme kinetics of TesA. The hydrolysis of 1-hexyl-glycerophosphocholine (C6-GPC), 1-lauryl-glycerophosphocholine (C12-GPC) and 1-palmitoyl-glycerophosphocholine (C16-GPC) follow Michaelis-Menten kinetics. Released fatty acids were quantified after 30 min incubation of 1 µg of purified TesA with 100 µl of lysoPL substrate at 37°C. (B) Subcellular localisation of TesA in *P. aeruginosa* PA01. Zymogram indicating esterase activity of cell compartments (S: supernatant, M: membrane fraction, Pp: periplasm fraction, Cy: cytoplasmic fraction) from *P. aeruginosa* PA01 transformed with pBBR-TesA (TesA) and pBBR1mcs-3 (EV, empty vector). Molecular weights of protein standard in kDa are indicated on the right. Esterase activity was monitored under UV light using the fluorescent substrate 4-methylumbelliferyl butyrate.

Our previous functional analysis of TesA revealed its pronounced hydrolytic activity against esterase substrates, but very low or no activity against phospholipase, thioesterase and protease substrates [12]. It was reported that *E. coli* TAP, which is highly similar to TesA, shows low catalytic activity towards lysophospholipid substrates [34,35]. We analysed the lysophospholipolytic activity of TesA using 1-acyl glycerophosphocholine (GPC) substrates of different acyl chain lengths, namely hexyl- (C6-GPC), lauryl- (C12-GPC) and palmitoyl- (C16-GPC) (Figure 1A). TesA was able to hydrolyse all the substrates tested (Table 1). The kinetic analyses of TesA with these substrates showed Michaelis-Menten kinetics with catalytic efficiencies (*k*_cat_/*K*_m_) for the hydrolysis of C6-GPC, C12-GPC and C16-GPC of 1.1 x 10^5^, 1.1 x 10^6^ and 4.5 x 10^5^ M^-1^ s^-1^, respectively. Such high values compared with the catalytic efficiency for the hydrolysis of the artificial substrate *p*-NP butyrate (12 x 10^3^ M^-1^ s^-1^) strongly suggest that lysophospholipase A activity may represent at least one of the physiological functions of TesA. Notably, lysophospholipids (lysoPLs) with twelve and sixteen carbon atoms are predominantly found in biological membranes, thus further supporting this assumption.

**Table 1:** Enzyme kinetics of TesA.

	**C6-GPC**	**C12-GPC**	**C16-GPC**
***K*_m_ (mM)**	7.22 ± 0.92	1.78 ± 0.17	2.04 ± 0.18
***k*_cat_ (s^-1^)**	1016.1	1975.3	921.1
***k*_cat_ / *K*_m_ (M^-1^ s^-1^)**	1.1 x 10^5^	1.1 x 10^6^	4.5 x 10^5^

Kinetic parameters (± standard deviation) for the hydrolysis of C6-GPC, C12-GPC and C16-GPC were determined by non-linear regression analysis of data fitted to the Michaelis-Menten equation.

Cellular localisation studies of TesA were performed in the homologous host *P. aeruginosa* PA01. A sequence-based prediction revealed a 21 amino acid long putative signal sequence in TesA suggesting its periplasmic or extracellular localisation. We expressed the *tesA* gene in *P. aeruginosa* using promoter P_*lac*_ of the broad host range vector pBBR1mcs-3 [36]. A well pronounced esterase activity band corresponding to a molecular mass of 20 kDa was detected in the periplasm of cells expressing TesA, but not in the extracellular fractions (Figure 1B Figure S2). These results indicate that TesA is primarily localized in the periplasm. As a control, *P. aeruginosa* PA01 wild type carrying the empty vector was used. A faint activity band was observed in the periplasm at ^~^ 20 kDa in case of control samples (Figure 1B Figure S2) as well as in *P. aeruginosa* PA01 wild-type grown overnight in LB medium (data not shown) which presumably represents constitutive expression of chromosomally encoded *tesA*.

### TesA exhibits an α/β/α-fold and a conserved GDSL hydrolase active site

**Figure 2:**
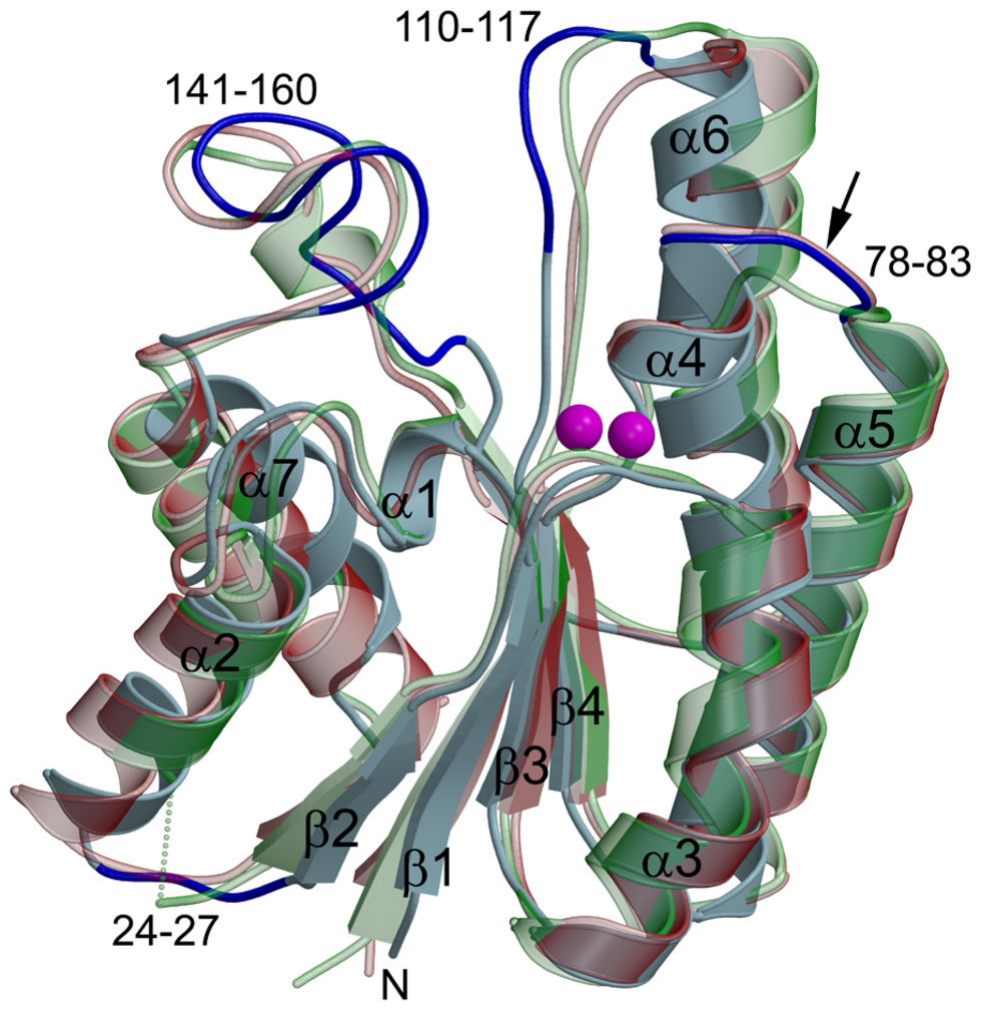
Crystal structure of TesA. Superposition of the overall structures of TesA (light blue); TAP (PDB code 1IVN, transparent green, Q=0.731) and EstA (PDB code 3HP4, transparent red, Q=0.743). Secondary structural elements and loops are labelled according to the TesA sequence. Two conserved water molecules (in PDB file Wat502 *rt* and Wat506 *lft*) in the TesA structure are shown as magenta balls. The loops which are structurally different from TAP and EstA are indicated in dark blue in the TesA structure. Note the narrow entrance of the catalytic site (between loop_110-117_ and loop_141-160_) in TesA structure. The black arrow indicates the position of the switch loop.

The crystal structure of TesA has been determined at 1.9 Å resolution. There are two molecules per asymmetric unit which are related by a two-fold noncrystallographic symmetry (NCS) (the root-mean-square difference (rmsd) between NCS related molecules A and B is 0.38 Å or Q=0.984; note: for identical structures Q=1). The monomer atomic model comprises residues 1-180 of the protein (Figure 2, light blue), no electron density was observed for the C-terminal His-tag comprised of residues 181-186. According to the Ramachandran plot, the model exhibits good geometry with none of the residues in the disallowed region (Table 2) [37].

**Table 2:** Data collection and refinement statistics of TesA.

Wavelength (Å)	0.9334
Resolution range (Å)	45.49 - 1.9 (1.93 - 1.9)
Space group	C 1 2 1
Unit cell	a=81.97 Å b=103.08 Å c=45.89 Å β=97.57°
Total reflections	93834
Unique reflections	29422
Multiplicity	3.2 (3.2)
Completeness (%)	98.90 (98.90)
I/sigma(I)	13.00 (4.70)
Wilson B-factor (Å^2^)	13.45
R-sym	0.064 (0.242)
R-factor	0.2268
R-free	0.2623
Number of atoms	2895
Protein residues	360
Water molecules	276
RMS (bonds) (Å)	0.007
RMS (angles) (Å)	1.02
Ramachandran favoured (%)	97
Ramachandran outliers (%)	0
Clash score	8.36
Average B-factor (Å^2^)	16.10

Statistics for the highest-resolution shell are shown in parentheses (resolution range 1.93 to 1.9 Å)

#### Overall Structure

The overall fold of TesA is similar to previously determined structures of enzymes belonging to the GDSL lipase family with a characteristic α/β/α-fold [6,9–11]. The compact single domain structure is comprised of a central five-stranded β-sheet surrounded by five α-helices and three 3_10_ helices. The rmsd values among 173/175 equivalent Cα atoms in our TesA structure and PDB codes 3HP4 (Figure 2, red) and 1IVN (Figure 2, green) are 1.5/1.58 Å (Q=0.743/0.731). Mainly, these differences can be assigned to the flexible loops comprising residues 34-37, 110-117 and 141-160 with the latter two loops forming part of the substrate binding site (Figure 2, shown in blue).

**Figure 3:**
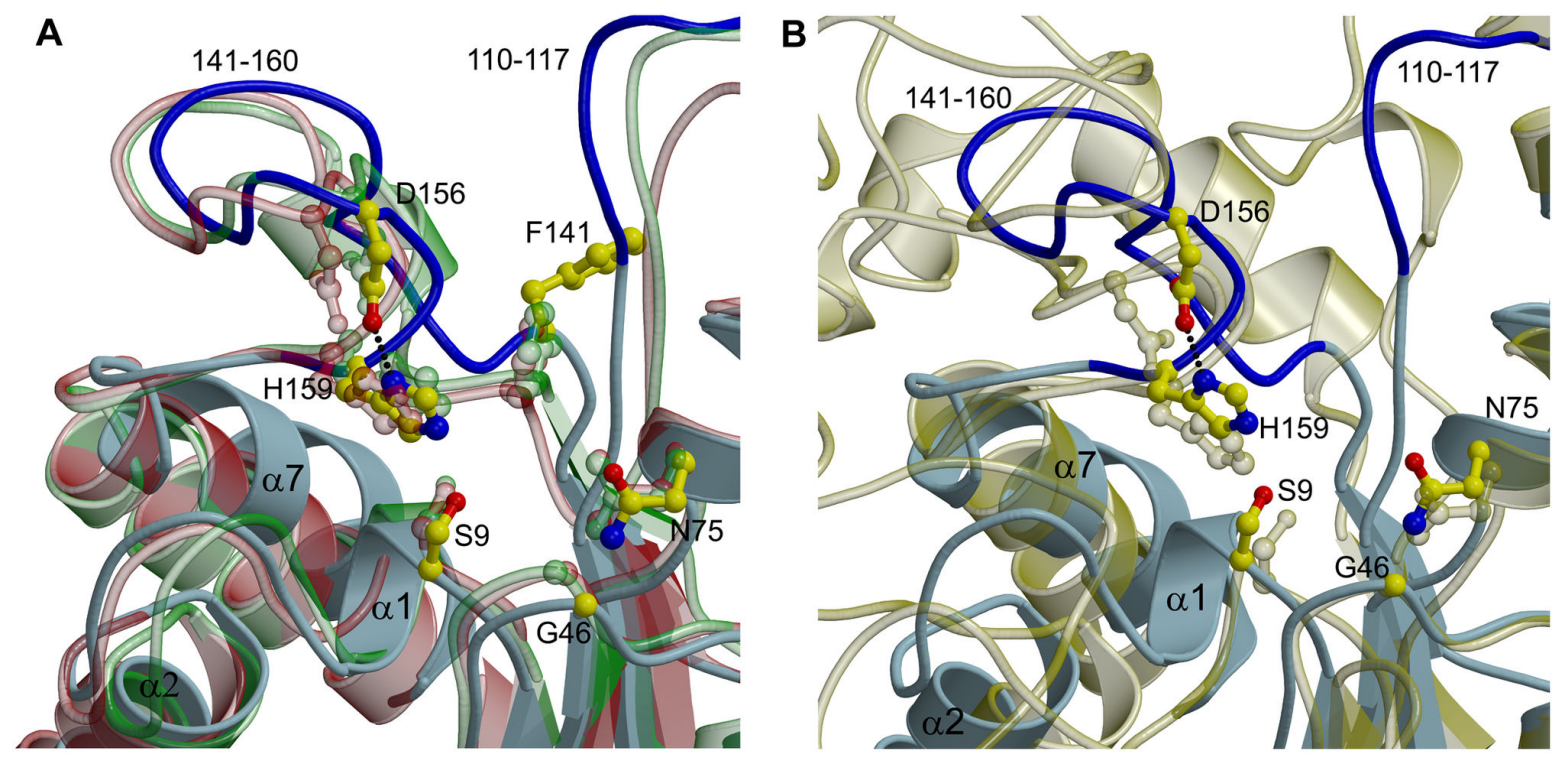
The catalytic site. (A) Superposition of TesA, TAP and EstA (colour labelling as in Figure 2). (B) Superposition of TesA and the catalytic domain of EstA of *P. aeruginosa* (PDB code 1KVN, transparent yellow). The catalytic residues (S9, H159, and D156) as well as residues forming the oxyanion hole (G46, N75) are shown as stick models. In the TesA structure, these residues are coloured by element with carbon in yellow, nitrogen in blue and oxygen in red. The dotted line represents the hydrogen-bond between the catalytic residues D156 and H159 in the TesA structure.

Based on sequence homology, we previously predicted Ser9, Asp156 and His159 as the active site catalytic triad residues, as well as residues Gly46 and Asn75 forming the oxyanion hole [12]. The structure of TesA confirmed these residues as active site residues located on the top of the solvent exposed substrate binding cleft (Figure 3). Both the positioning and the orientation of the catalytic triad residues at structurally conserved topological sites classify TesA as a typical GDSL hydrolase.

#### Comparison of TesA and related enzyme structures

A DALI search [38] with the TesA structure revealed 27 best hits that were all GDSL-hydrolases with Z-scores higher than 12. The Z-score is a measure of structural similarity that exceeds a value of 2 for protein structures with similar folds. In general, TesA shows low sequence homology to other GDSL hydrolases (7–27% identity) with the exceptions of TAP from *E. coli* [7] and EstA [9] from the evolutionarily related bacterium *Pseudoalteromonas* sp. 643A which show 49% and 42% sequence identity, respectively. Not surprisingly, the respective structures were also identified as the best hits in the DALI database search with Z-scores of 26.8 (TAP, 1IVN) and 28.3 (EstA, 3HP4). Compared to all other GDSL hydrolases, the catalytic domain of the autotransporter esterase EstA from *P. aeruginosa* [10] shows least structural similarity with TesA (rmsd = 3.21 Å and Q = 0.195 for 151 residues), mainly due to low sequence similarity and a 29-residue insertion in block IIIa of the EstA sequence. However, superposition of the two structures reveals a similar fold and a similar orientation of the active site residues (Figure 3B).

Although a comparison of several GDSL hydrolase structures with TesA revealed a similar fold, they differ significantly in their enzymatic properties. We have previously reported enzyme promiscuity in several GDSL hydrolases by testing them against four chemically different classes of substrates known to be hydrolysed by esterases, thioesterases, lipases and phospholipases, respectively [12]. TesA, for example, differs from its closest neighbour TAP as it shows very little thioesterase activity, no protease activity, and an esterase activity with a preference for *p*-nitrophenyl ester substrates with short- and mid-range carbon chain length (C4-C8). In contrast, TAP is a multifunctional enzyme that shows esterase, thioesterase, protease and lysophospholipase A activities [39]. Despite these functional differences, the rmsd among 173 equivalent C_α_ atoms in TesA and TAP (1IVN) structures is 1.58 Å (Q=0.731). Assuming that functional differences regarding substrate specificity may reflect structural differences between TesA and TAP in the loops located at the substrate binding site (Figure 3A), we subjected these parts of the respective structures to a more detailed comparison.

In previous reports, Liaw and colleagues have reported several structures of TAP in the apo (unbound) state [7], and in the presence of ligands [39]. A structural comparison revealed two different conformations in the switch loop formed by amino acids 75-80 that is structurally equivalent to loop_78-83_ in TesA. The authors argue that this conformational change is induced by hydrophobic interactions upon binding of the octanoic acid (OCA) molecule, and is acyl chain length dependent [39]. Interestingly, the conformation of the respective loop in the apo (i.e. ligand-free) form of the TesA structure is similar to that in the complex of TAP with the substrate OCA, but not to the TAP apo state. Lo et al. have previously argued that the switch loop movement is dependent on the length of the acyl chain of the ligand molecule. This conclusion was based on the crystal structures of TAP complexes where the loop movement takes place in the presence of OCA, but not with bound inhibitor, diethyl *p*-nitrophenyl phosphate (DEP) with an acyl chain length of 2 carbon atoms [7]. In contrast, in the previously reported structure of *Pseudoalteromonas* EstA bound to the inhibitor monoethyl *p*-nitrophenyl phosphate [9], the conformation of the switch loop is similar to TAP-OCA (long acyl chain length) and not to TAP apo state or TAP-DEP (short acyl chain length).

Taken together, TesA and previously reported crystal structures show that the switch loop (indicated with an arrow in Figure 2) adopts two conformations. However, there is no strict correlation between a preferred conformation and the presence of a ligand as has been suggested earlier for TAP [39]. This conclusion is further supported by the observation that residues in the switch loop in all GDSL hydrolase structures are not in close contact with the catalytic triad and the oxyanion hole and are thus not involved in catalysis and in stabilisation of the transition state intermediate. Nevertheless, the dynamics of the switch loop might have an important role in substrate binding most likely *via* an altered hydrophobic surface of the substrate binding crevice as in case of TAP [39].

#### TesA reveals a compact and rigid structure

**Figure 4:**
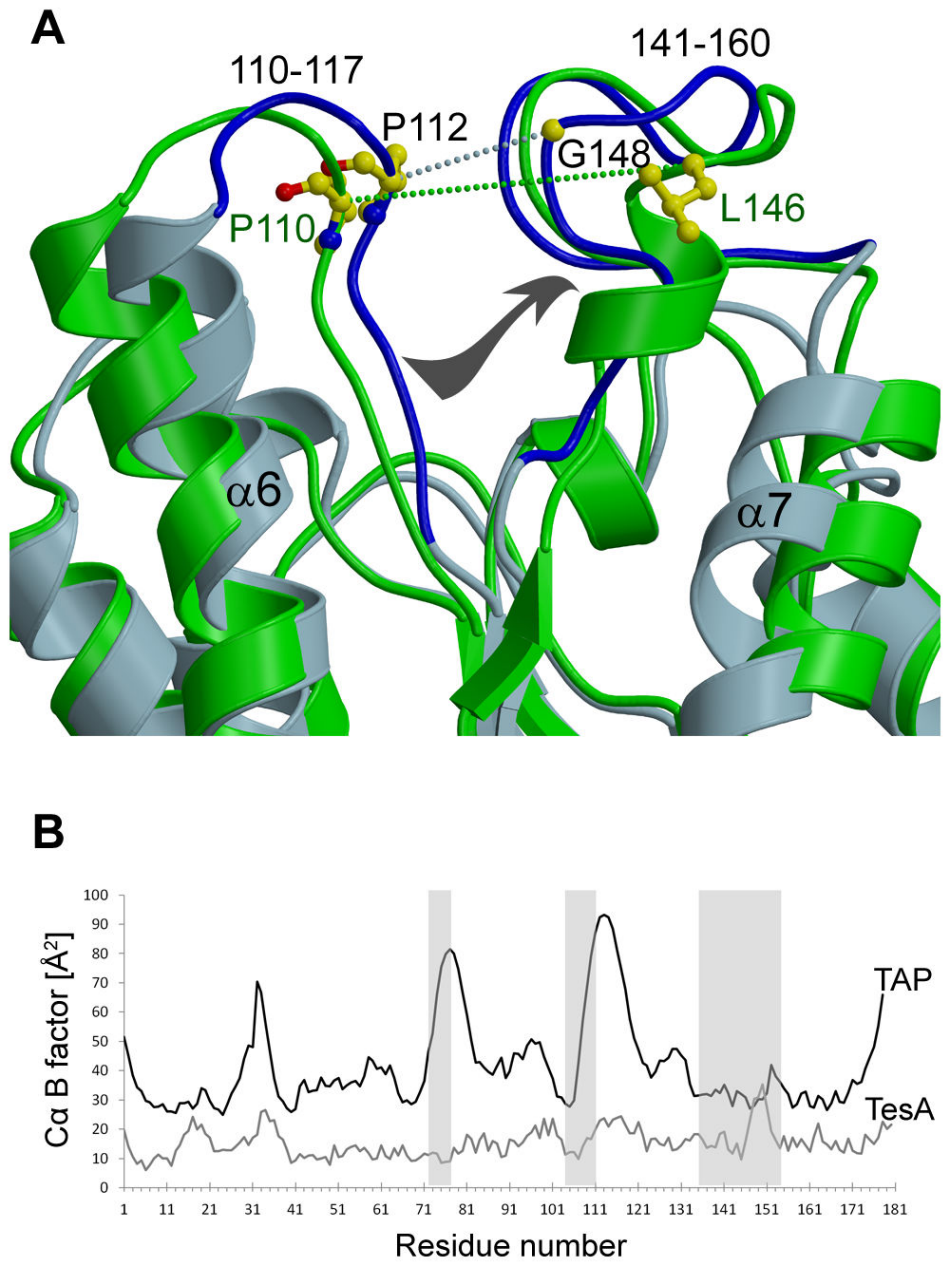
TesA reveals a compact and rigid structure. (A) Comparison of the crystal structures of TesA (light blue) and TAP (green). Note the positioning of loop_110-117_ and loop_141-160_ (both loops shown in dark blue) in TesA at the entrance of the substrate binding cavity, and the shift of helix α7 towards the core. Dotted lines represent the interatomic distances between Cα atoms of residues Pro112 and Gly148 in TesA, and equivalent residues Pro110 and Leu146 in TAP that are 6.1 Å and 13.6 Å, respectively. (B) Plot of average temperature factor of the protein backbone versus residue of TesA and TAP (PDB ID: 1IVN). Switch loop_78-83_, loop_110-117_ and loop_141-160_ are indicated as shaded regions.

In TesA, helix α6 and loop_119-140_ (connecting β5 and α8) located at the entrance of the substrate binding cavity move closer towards the cleft (Figure 4A). The interatomic distances between Cα atoms of residues Pro112 and Gly148 in TesA, and equivalent residues Pro110 and Leu146 in TAP are 6.1 Å and 13.6 Å, respectively. Consequently, the adjacent helices α7 and α8 in TesA are also shifted towards the protein core, forming a more compact structure. The B- (or temperature) factor is an important parameter that reflects flexibility in a protein crystal structure, as it indicates thermal motion or disorder. Flexibility in the three loops important for substrate binding and hydrolysis was previously reported in all TAP structures [13]. Interestingly, a comparative B-factor analysis of TesA and TAP revealed significantly lower values for TesA (Figure 4B). The more compact structure and the lower B-factor values clearly indicate pronounced conformational rigidity of TesA. This, in turn, might exert an influence on the substrate specificity of the enzyme. Introduction of point mutation L109P in loop_109-120_ located near the active site cleft in TAP resulted in increased rigidity of the active site *via* formation of additional hydrogen bonds as seen in the crystal structure and from the B-factor analysis [13,39], whereas no other structural differences were observed in this TAP variant. Additionally, when compared to wild-type TAP, variant L109P showed 7.2- and 10-fold lower efficiency to hydrolyse long acyl chain substrates palmitoyl-CoA (C16, thioester) and *p*-nitrophenyl dodecanoate (C12), respectively [13]. These results suggest that minor changes in the active site can result in significant alterations in substrate specificity, without affecting the enzyme’s tertiary structure. TesA shows a more rigid, compact structure with low thioesterase activity, and hence a preference for *p*-nitrophenyl ester substrates with mid-range carbon chain length (C4-C8). Apparently, GDSL hydrolases possess a flexible active site that undergoes conformational rearrangements upon substrate binding, thus following an induced-fit mechanism [2]. Consequently, parameters like the flexibility of substrate binding loops play an important role in defining the geometry and physico-chemical properties of the active site pocket that is directly related to the substrate specificity of GDSL hydrolases. A similar observation was reported for the short-chain dehydrogenase/reductase and the amidohydrolase families of enzymes in which the diversity of loop conformations in the substrate binding domains result in changes in the shape and size of the active sites, a property that allows hydrolysis of a broad range of substrates by these enzymes [40,41]. Calmodulin represents a typical example for loop flexibility resulting in protein promiscuity due to changing physico-chemical properties of the substrate binding site. Calmodulin can bind different target proteins due to conformational changes of surface-exposed flexible loops, apparently exposing hydrophobic patches responsible for substrate recognition [42].

#### Residues involved in the formation of substrate binding cavity are structurally conserved amongst GDSL hydrolase family

The catalytic triad residues of TesA were found to be structurally identical to those in TAP and *Pseudoalteromonas* EstA. We next examined the substrate binding cavities in TesA and compared them to previously reported structures of GDSL hydrolases using the program VOIDOO [43]. Among all 39 structures evaluated, cavities were detected only in the substrate binding clefts of TesA, *P. aeruginosa* EstA (1KVN) and the TAP-OCA complex (1U8U) (Figure S3). A comparison of the amino acid residues lining the surface of these channels revealed a similar composition in TesA and TAP (see Table S2 for a complete list). The catalytic triad residues and the residues lining the cleft on the inner side are identical with similar side chain conformations. However, some variations are seen at the cleft entrance in the loops, for example, structural equivalents of residues Pro113 and Met153 are not found in the channel of TAP. Structural superposition revealed that these residues reside in the loops (loop_110-117_/α6, loop_141-160_) that move closer to the cleft in case of TesA (Figure 4A). Interestingly, the corresponding loops show structural variations, modulating the width of the substrate binding cleft, with TesA showing the narrowest and EstA the widest entrance of the cleft region (Figure 4A). Another noticeable difference is seen between residue Phe141 of TesA and the respective structurally equivalent residues Phe139 and Phe143 in TAP and EstA, with the former showing a side chain conformation rotated outwards (Figure 3A) and the latter two pointing towards the cleft. The conformation of Phe139 in the TAP apo-as well as ligand-bound structure is identical suggesting that the presence of ligand has no effect on the side chain conformation of this residue. Phe141 in TesA is located on loop_141-160_ that shows very low sequence similarity to other GDSL hydrolases (Figure S1). Additionally, the functional significance of his loop is obvious as it contains two members of the catalytic triad namely Asp156 and His159, at the C-terminal end (Figure 3A). Such differences in the residues lining the substrate binding cleft could have an impact on the fine tuning of enzyme activity *via* favourable or unfavourable interactions with specific substrates.

#### Role of two conserved water molecules

**Figure 5:**
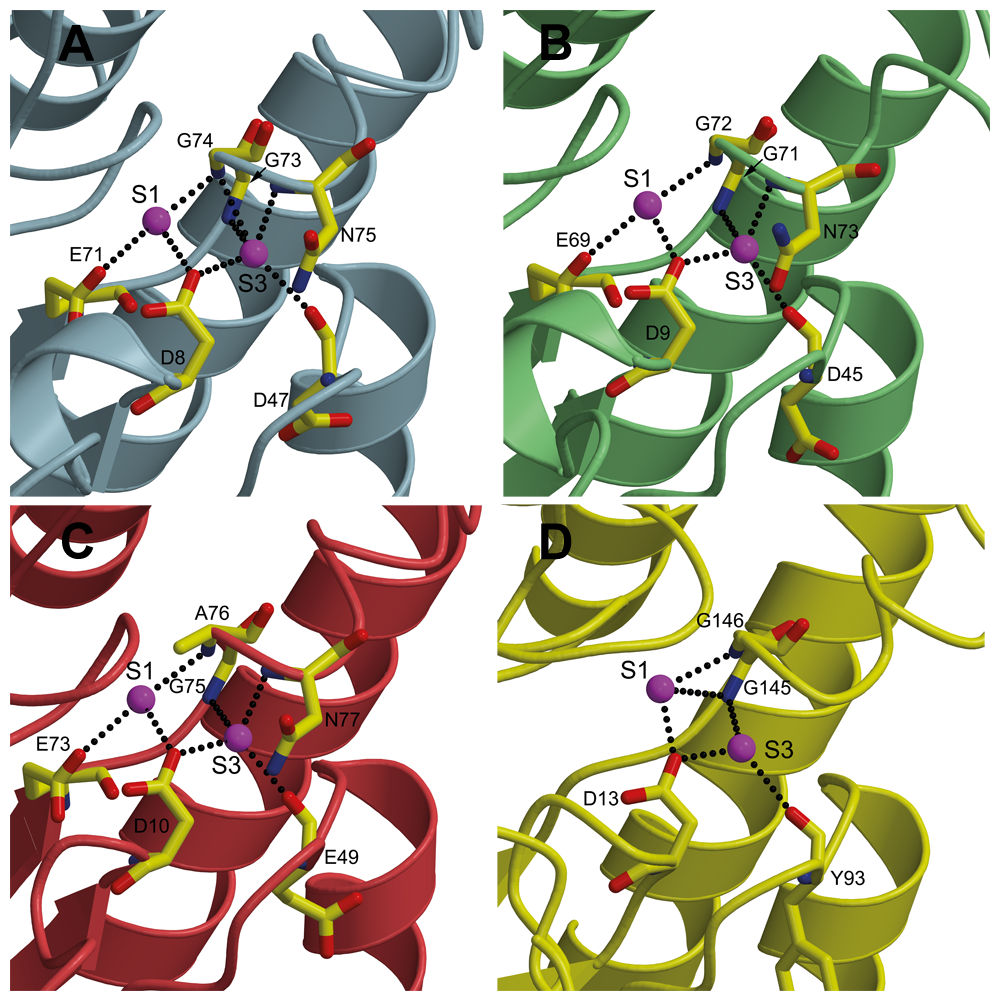
Conserved waters and hydrogen bonds in the catalytic site. (A) TesA, (B) TAP (PDB ID: 1IVN), (C) EstA (PDB ID: 3HP4), and (D) esterase domain of the autotransporter EstA (PDB ID: 3KVN). The hydrogen bonds mediated by the two conserved water molecules (S1 and S3 shown as magenta balls) are shown as dotted lines.

A conserved hydrogen bond network has been previously reported for other GDSL hydrolases [7]. Structurally conserved water molecules usually stabilize the positions of residues and fold through hydrogen-bonding. We examined the TesA crystal structure for the presence of previously identified conserved waters S1 and S3 in TAP and other GDSL hydrolases [7]. The structural alignment shows the presence of both waters in TesA, in identical spatial positions and with the protein environment comprised primarily of highly conserved residues. The S1 water molecule is hydrogen-bonded to OD2 atom of Asp8, OE2 of Glu71, and the amide N of Gly74 (Figure 5). The S3 water is hydrogen bonded to OD2 of Asp8, O of Asp47, and the amide nitrogens of Gly73, Gly74 and Asn75, respectively. The protein residues involved in this hydrogen bond network are located in the conserved amino acid sequence blocks I, II and III of the GDSL hydrolase family (Figure S1). Moreover, they are either part of the oxyanion hole itself or are in close proximity to residues forming the oxyanion hole and to the catalytic Ser9. It is thus likely that the conserved waters serve a structural as well as catalytic function by positioning the catalytically important residues in the unique fold observed for structurally conserved enzymes of the GDSL hydrolase family.

#### Enhanced thioesterase activity of TesA by rational mutagenesis

Despite the notable structural similarity between *P. aeruginosa* TesA and *E. coli* TAP, their thioesterase activities differ significantly with TesA exhibiting only one third of the thioesterase activity of TAP as measured with the substrate palmitoyl-CoA (C16-CoA). For TAP, amino acids Ser18 and Arg160 were proposed as residues involved in binding of the CoA substrate *via* hydrogen bonding (Ser18) and ion-ion interactions (Arg160) with the negatively charged phosphate moieties of CoA [39]. The structurally equivalent residues in TesA are Asp17 and Leu162, respectively. Reasonably, one could assume that the negatively charged Asp17 and the non-polar Leu162 of TesA might not be favourable for interactions with the negatively charged phosphate groups of CoA.

**Figure 6:**
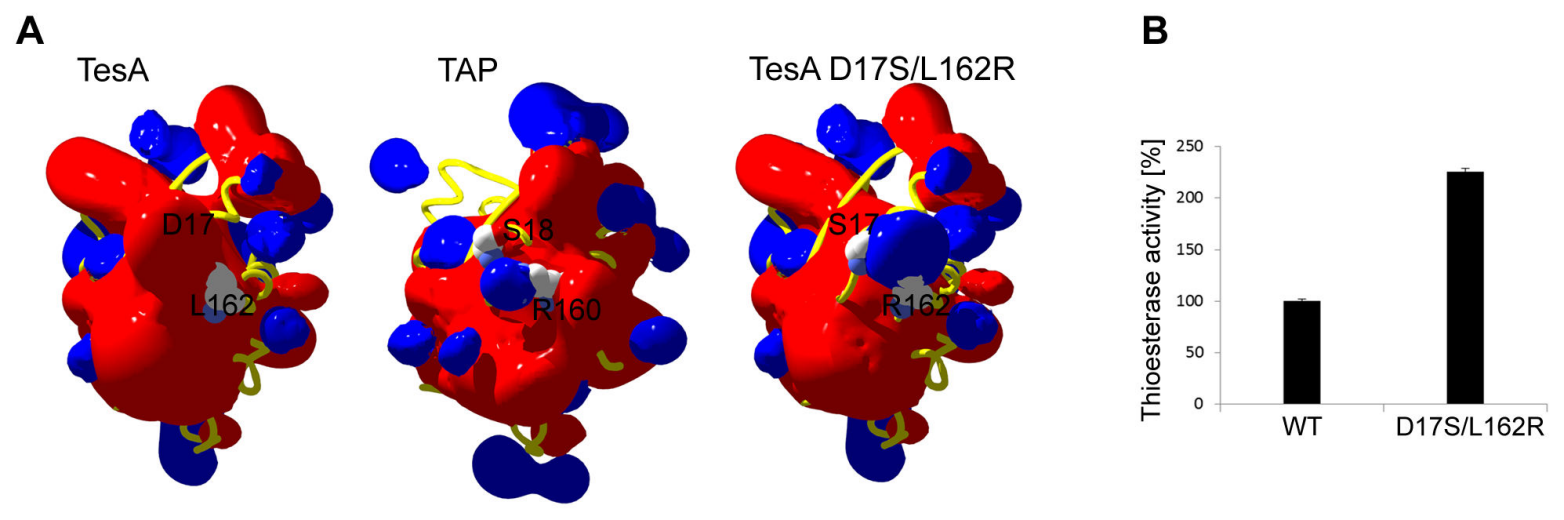
Rational mutagenesis of TesA. (A) Electrostatic potential maps around TesA, TAP and variant TesA D17S/L162R showing the field that propagates into the solvent. Cutoff values of +1.8 kT/e and -1.8 kT/e were used to define the blue and red contours, respectively. Note the positive potential localized at R160 in TAP (structural equivalent L162 is indicated in TesA), and R162 in variant TesAD17S/L162R). The orientation of molecules is as in Figure 2. (B) Enhanced thioesterase activity of TesA. Thioesterase activity assays were performed using lauroyl-CoA (25 µM) as a substrate with purified his-tagged TesA (1 µg); activity of the wild type TesA (WT) was taken as 100%. The results are mean values of three independent measurements with standard deviation indicated by error bars.

Figure 6A illustrates the electrostatic potential maps around TesA, TAP and the TesA double mutant (TesA D17S/L162R). In the latter case, however, calculations were performed on the TesA crystal structure where the two relevant residues were first mutated using the most favoured rotamer in the program COOT [28]. The figure therefore shows the predicted electrostatic potential map for the double mutant. Both, TesA and TAP show mostly negative potential around the respective core domains. A positive patch specific to TAP is localized at Arg160 (the structural equivalent Leu162 in TesA is indicated). As postulated, the double mutant TesA D17S/L162R shows a positive potential around residue 162.

These observations suggest that a change in the electrostatic potential of the CoA binding pocket may affect thioesterase activity of TesA. We thus mutated residues Asp17 and Leu162 to Ser and Arg, respectively. The purified mutant protein TesA D17S/L162R was used to determine the thioesterase activity using lauryl-CoA (C12-CoA) as the substrate. As shown in Figure 6B, in comparison to the wild-type TesA, thioesterase activity of the double mutant increased by 2.2-fold. Apparently, TesA serves as an example of how compact proteins evolve by a few amino acid exchanges to exert different enzymatic functions.

### Putative physiological function of TesA

Both sequence analyses and the crystal structure reveal that TesA belongs to the conserved GDSL hydrolase family. Members of the GDSL hydrolase family of serine lipases possess a broad substrate specificity and regiospecificity [2]. We previously reported high arylesterase, minor thioesterase and no protease activities for TesA [1]. In the present work, we show that TesA shows lysophospholipase A (lysoPL A) activity with substrate specificity for medium and long chain lysophospholipids (lysoPLs), which are predominantly present in the bacterial cell membranes [44,45]. Furthermore, we show that TesA is localised primarily in the periplasm of *P. aeruginosa* PA01, enabling access to phospholipids present both in the outer and the inner membranes (Figure 1B Figure S2). Wild-type *P. aeruginosa* PA01 also produces catalytically active TesA at a low basal level indicating a housekeeping function for this enzyme. In addition, *P. aeruginosa* TesA is evolutionary conserved among pathogenic as well as non-pathogenic *Pseudomonas* species (Figure S4 and Table S3) suggesting an important physiological function, e.g. for membrane phospholipid homeostasis. Indeed, low binding affinities and high catalytic efficiencies for lyso-GPC substrates, with *K*_m_ values in the mM range, may point to TesA functioning in the regulation of lysoPL levels. Presumably, TesA binds lysoPLs which may be located either in the inner leaflet of the outer membrane and the outer leaflet of the inner membrane, followed by their rapid hydrolysis to bring down the concentration of lysoPLs to desired physiological levels. Although the lysoPL content in the membranes of *P. aeruginosa* is currently unknown, a huge variation (of the lysoPL content) between 2% and 50% in the membranes of other Gram-negative pathogenic bacteria has been reported [46].

Living organisms adapt their membrane lipid composition as a response to different environmental and physiological conditions [47]. In particular, lysoPLs affect the biophysical properties of membranes and influence the functions of membrane-embedded proteins [48]. They are related with several important processes in eukaryotes such as neurotransmitter release [49], regulation of the membrane fluidity [50], and phagocytosis [51]. Limited data exist for their function in bacteria; few examples include the *E. coli* diacylglycerol kinase, which is a homotrimeric integral membrane protein that is stabilised by the presence of lysoPLs [52]. The mechanosensititve channels MscL and MscS of *E. coli*, which play a protective role under the conditions of osmotic shock, are activated by lysoPLs [53]. One of the best studied bacterial enzymes is the extracellular lysophospholipase PlaA from the pathogenic bacterium *Legionella pneumophila* [21], which plays a role in detoxification of exogenously added lysoPLs that are cytotoxic for *L. pneumophila* [21]. Interestingly, a sequence homolog (orf PA2927, 25% protein sequence identity) of PlaA from *L. pneumophila* [20] is found in *P. aeruginosa* PA01, too. Hence, we performed similar experiments with *P. aeruginosa* PA01 and observed that this strain is not susceptible to exogenously added lysoGPC, at least at a concentration of 0.2 mM (data not shown). Moreover, lysophospholipase A activity was detected predominantly in the periplasm and membrane fractions of *P. aeruginosa* PA01 (Figure S5). Our observations suggest that TesA, presumably together with a still unknown membrane-bound lysoPL, may accomplish detoxification of exogenously added lysoPLs. The regulation of bacterial membrane fluidity by altering the amount of phospholipids with unsaturated fatty acids represents another potential function of lysophospholipase A. This phenomenon known as “homeoviscous adaptation” is common among bacteria [54–56]. Such an adaptation based on activity changes of fatty acid biosynthesis enzymes was reported for *Pseudomonas* *putida* [57] and for other bacteria [58]. However, in bacteria virtually nothing is known about the regulation of membrane fluidity by hydrolysis of phospholipids. Here, we have explored whether TesA might participate in membrane fluidity regulation through the specific hydrolysis of lysoPLs with bound unsaturated or saturated fatty acids. TesA showed twice the activity towards oleoyl-lysoPL (unsaturated) *versus* stearoyl-lysoPL (saturated) (Figure S6) suggesting that TesA might be involved in regulation of the ratio of saturated to unsaturated fatty acids in *P. aeruginosa* membrane lipids, participating in environmental adaptation. Thus, TesA may be part of a complex enzymatic system responsible for phospholipid homeostasis in the opportunistic human pathogen *P. aeruginosa*.

## Supporting Information

Table S1 Strains and plasmids used in this study. (PDF)

Table S2 Comparison of the amino acid residues lining the surface of channels in TesA, TAP and EstA. (PDF)

Table S3 Orthologs of *P. aeruginosa* TesA from genus *Pseudomonas* . (PDF)

Figure S1 Structure based sequence alignment of TesA. Note the high structural conservation in the regions embracing the catalytic amino acids (blocks I, II, III and V). TAP, (PDB ID: 1IVN), thioesterase from *E. coli* [7]; EstA, (PDB ID: 3HP4), esterase from *Pseudoalteromonas* *sp*. Identical and similar amino acids are shaded in black and grey, respectively. Catalytic triad residues of TesA and oxyanion hole residues are indicated in yellow and red, respectively. The asterisks (*) represent residues interacting with conserved water molecules and black dots (●) represent residues which show enhanced thioesterase activity after mutations in TesA. Underneath the sequence alignment are shown secondary structure elements of TesA.
(PDF)

Figure S2 Positive controls for Western blot analysis. Antibodies used are against the periplasmic protein DsbA, the outer membrane protein XcpQ and the extracellular protein ToxA. The gel contained equivalent amounts of the membrane (Me), cytoplasmic (Cy), periplasmic (Pp) proteins, and a three-fold excess of extracellular proteins isolated from culture supernatant (Su). For Western blotting, proteins were electrophoretically transferred from the SDS-gel to a polyvinylidene difluoride (PVDF) membrane using a Mini Trans-Blot® Electrophoretic Transfer Cell (BioRad) following the manufacturer recommendations. XcpQ, ToxA and DsbA were detected by incubating the membranes with specific polyclonal antibodies diluted 1:5000, 1:5000, or 1:50000, respectively, with TBST buffer (0.1 M Tris-HCl, 0.1 M NaCl, pH 7.5, Tween 20 0.5% v/v), followed by an incubation with anti-rabbit IgG-horseradish peroxidase conjugate antibodies (Bio-Rad). The blots were developed with an ECL Western blotting detection kit (GE Healthcare).
(PDF)

Figure S3 Substrate binding cavities. Residues involved in the formation of substrate binding cavities and channels in the GDSL family hydrolases TesA, Tap, and EstA calculated using program VOIDOO [43]..
(PDF)

Figure S4 Sequence alignment of orthologs of *P. aeruginosa* TesA from genus *Pseudomonas* . Details of sequences are given in Table S3. Identical and similar amino acids are depicted on black and gray background.
(PDF)

Figure S5 Distribution of lysophospholipase activity in *P. aeruginosa* PA01. Lysophospholipase A activity was measured with C16-PGC as the substrate. A *P. aeruginosa* PA01 culture grown in LB medium at 37°C to stationary phase was used for cell fractionation. The assays were performed with 25 µL of fractions isolated from one ml of cell culture with OD_580nm_ of 1. The relative activities were calculated by dividing the absolute activity of each fraction by the total lysophospholipase activity detected in cell extracts of *P. aeruginosa* PA01.
(PDF)

Figure S6 Lysophospholipase A activity of TesA measured with saturated and unsaturated lysophospholipids. In the assay, 1 µg of purified TesA and 0.67 mM substrate (1-stearoyl-glycerophosphocholine C18-GPC, or 1-oleoyl-glycerophosphocholine, C18: 1-GPC) was used as described in Methods section. Activity of TesA with C18: 1-GPC was taken as 100%.
(PDF)
