# Supplementary material for: Structural and Functional Characterisation of TesA - A Novel Lysophospholipase A from Pseudomonas aeruginosa
Source: PLoS One. 2013 Jul 18;8(7):e69125. doi: 10.1371/journal.pone.0069125 (PMC3715468; doi:10.1371/journal.pone.0069125)
Supplement: Table S1 — (PDF) [file pone.0069125.s001.pdf]

**Strains**	**Genotype**	**Source**
E. coli DH5α	*supE44* Δ(*lacZYA-argF*)*U196* (*Φ80*Δ*lacZM15*) *hsdR17 recA1 endA1 gyrA96 thi-1 relA1*	[1]
E. coli BL21(DE3)	F$^-$ *ompT hsdSB*($r_B^- m_B^-$) *gal dem* (*λIts*857 *indI Sam*7 *nin*5 *lavUV*5-T7*gene*1)	[2]
E. coli S17.1	Ec294:: [RP4-2(Tc::Mu)(Km::Tn7)]<br>Tp^r, Sm^r, *tra*$^+$ *recA, pro, res*	[3]
P. aeruginosa PA01	wild type<br>originating from Dieter Haas laboratory (Lausanne, CH)	[4]
**Plasmids**	**Description**	**Source**
pET22b+	ColE1 PT7$_{\Phi 10}$ *pel*B Ap^r C-His$_6$-„Tag“ *lacI^q*	Novagen
pBBR1mcs3	Cm^r mob *lacZα Plac* PT7 Tc^r	[5]
pET22b-TesAH6	*tesAH6* gene inserted in *Nde*I/*Sac*I of pET22b(+)	[6]
pET22b-TesA	*tesA* gene inserted in *Nde*I/*Sac*I of pET22b(+)	this study
pBBR1mcs3-TesA	*Xba*I/*Sac*I fragment of pET22b-TesA inserted in pBBR1mcs-3	this study
pET22b-TesAH6$_{D38S_L183R}$	*tesA* gene with substitutions of Asp38 and Leu183 with Ser and Arg respectively inserted in *Nde*I/*Sac*I sites of pET22b(+)	this study
**Oligonucleotides**	**DNA Sequence (5'→3')**	**Features**
TesA_Up	TAACATATGCGTGCATTGCTG	*Nde*I
TesA_DW	TAAGAGCTCTAACTCGAGAAGCAGCGGTTTCAG	*Sac*I
TesA_D38S_Up	GCCGCTTTGGGACTGA^{G}G^{A}TACCAGCCAGGGCTG	Asp → Ser
TesA_D38S_Dw	CAGCCCTGGCTGGTACT^{C}C^{T}AGTCCCAAAGCGGC	Asp → Ser
TesA_L183R_Up	CATCCGGCGCG^{T}CGCCGCCCAG	Leu → Arg
TesA_L183R_Dw	GTAGGCCGCGC^{A}GCGGCGGGTC	Leu → Arg

References to Table S1

1. Woodcock DM, Crowther PJ, Doherty J, Jefferson S, DeCruz E, et al. (1989) Quantitative evaluation of *Escherichia coli* host strains for tolerance to cytosine methylation in plasmid and phage recombinants. Nucleic Acids Res 17: 3469-3478.
2. Studier FW, Moffatt BA (1986) Use of bacteriophage T7 RNA polymerase to direct selective high-level expression of cloned genes. J Mol Biol 189: 113-130.
3. Simon R, Priefer U, Pühler A (1983) A broad host range mobilization for *in vitro* genetic engeneering: transposon mutagenese in gram negative bacteria Bio Technol 1: 784-791.
4. Holloway B, W., Krishnapillai V, Morgan AF (1979) Chromosomal genetics of *Pseudomonas*. Microbiol Rev 43: 73-102.
5. Kovach ME, Phillips RW, Elzer PH, Roop RM, 2nd, Peterson KM (1994) pBBR1MCS: a broad-host-range cloning vector. Biotechniques 16: 800-802.
6. Lescic Asler I, Ivic N, Kovacic F, Schell S, Knorr J, et al. (2010) Probing enzyme promiscuity of SGNH hydrolases. Chembiochem : a European journal of chemical biology 11: 2158-2167.
