# Supplementary material for: Structural and Functional Characterisation of TesA - A Novel Lysophospholipase A from Pseudomonas aeruginosa
Source: PLoS One. 2013 Jul 18;8(7):e69125. doi: 10.1371/journal.pone.0069125 (PMC3715468; doi:10.1371/journal.pone.0069125)
Supplement: Table S2 — (PDF) [file pone.0069125.s002.pdf]

**TesA**	**TAP (PDB ID: 1U8U)**	**EstA (PDB ID: 3KVN)**
8-DSI-10	9-DSL-11	13-DSL-15
-	-	34-F (*14-L*)*
-	43-SG-44 (*45-SG-46*)*	91-GG-92 (*45-SG-46*)*
74-GN-75	72-GN-73	146-GN-147
78-L	76-LR-77 (*78-LR-79*)*	150-L
-	-	185-W
109-IQLPP-113	107-IRLP-110	187-LPDL-190
-	113-Y (*115-Y*)*	-
-	139-F (*141-F*)*	-
143-L	141-M	-
147-GG-148	145-Y	-
153-M	-	-
157-GIH-159	155-GIH-157	-
-	-	192-LT-193
-	-	232-I
-	-	235-LL-236
-	-	239-GM-240
-	-	246-F
-	-	248-L
-	-	283-LF-284
-	-	288-VHP-290
-	-	294-G
-	-	297-LI-298

*Residues in italics in parentheses are structural equivalents in TesA, which do not participate in the channel lining
