# Supplementary material for: Structural and Functional Characterisation of TesA - A Novel Lysophospholipase A from Pseudomonas aeruginosa
Source: PLoS One. 2013 Jul 18;8(7):e69125. doi: 10.1371/journal.pone.0069125 (PMC3715468; doi:10.1371/journal.pone.0069125)
Supplement: Table S3 — (PDF) [file pone.0069125.s003.pdf]

**Organism**	**Sequence identity***	**UniProtKB Acc**
P. pseudoalcaligenes	73 %	L8M832
P. mendocina	71 %	A4XVE8
P. protegens	71 %	Q4K8R3
P. putida	71 %	Q88KH2
P. chlororaphis	71 %	I4Y2Q8
P. syringae	70 %	Q4ZUR1
P. viridiflava	70 %	K6BJH0
P. synxantha	70 %	I4KT20
P. brassicacearum	69 %	F2K9A1
P. entomophila	69 %	Q1IC90
P. fluorescens	69 %	Q3K903
P. fulva	66 %	F6AFG5
P. stutzeri	62 %	F8H9H8

*Sequence homology search with the sequence of *P. aeruginosa* TesA was performed using BLAST tool on UniProt web site (http://www.uniprot.org)
