# Supplementary figures and images for: Structural and Functional Characterisation of TesA - A Novel Lysophospholipase A from Pseudomonas aeruginosa
Source: PLoS One. 2013 Jul 18;8(7):e69125. doi: 10.1371/journal.pone.0069125 (PMC3715468; doi:10.1371/journal.pone.0069125)

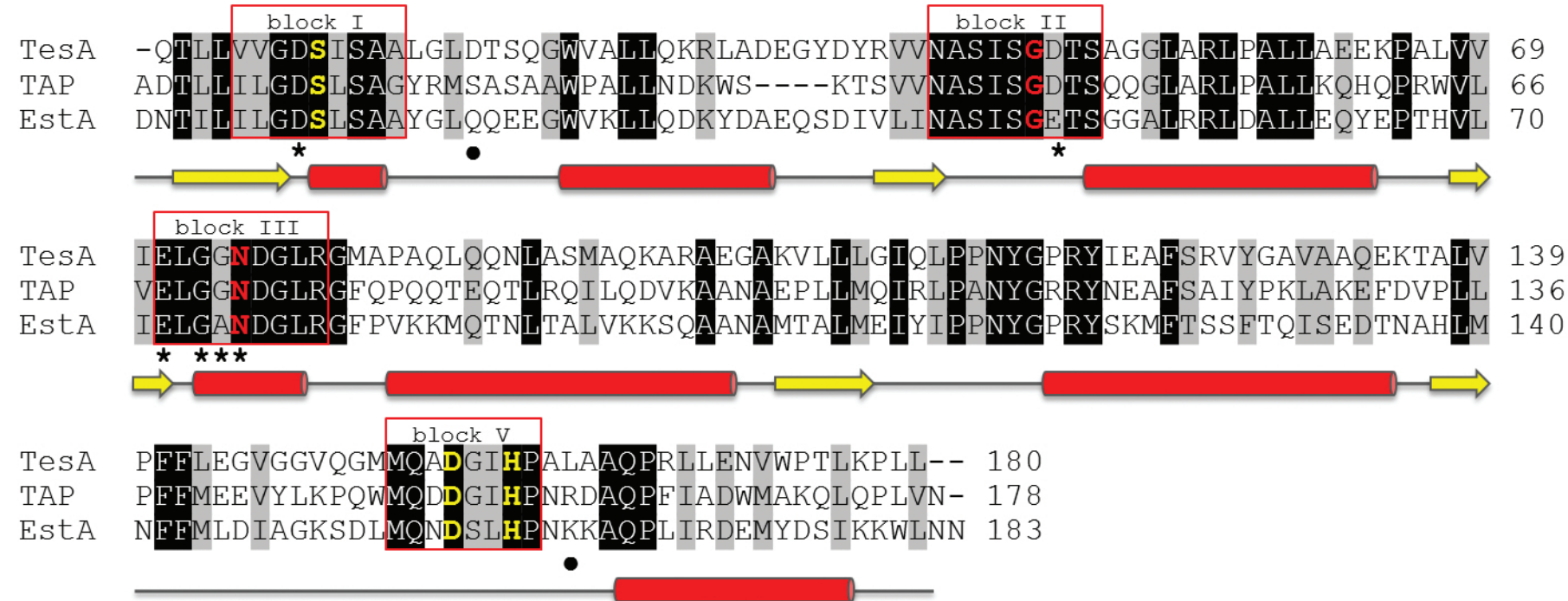

Supplement: Figure S1 — Note the high structural conservation in the regions embracing the catalytic amino acids (blocks I, II, III and V). TAP, (PDB ID: 1IVN), thioesterase from E. coli [7]; EstA, (PDB ID: 3HP4), esterase from Pseudoalteromonas sp. Identical and similar amino acids are shaded in black and grey, respectively. Catalytic triad residues of TesA and oxyanion hole residues are indicated in yellow and red, respectively. The asterisks (*) represent residues interacting with conserved water molecules and black dots (●) represent residues which show enhanced thioesterase activity after mutations in TesA. Underneath the sequence alignment are shown secondary structure elements of TesA. (PDF) [file pone.0069125.s004.pdf]

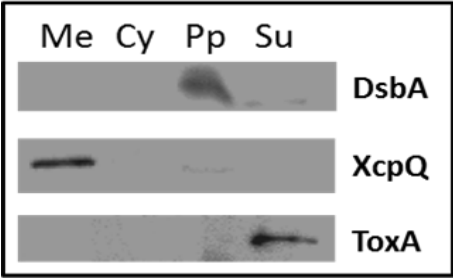

Supplement: Figure S2 — Antibodies used are against the periplasmic protein DsbA, the outer membrane protein XcpQ and the extracellular protein ToxA. The gel contained equivalent amounts of the membrane (Me), cytoplasmic (Cy), periplasmic (Pp) proteins, and a three-fold excess of extracellular proteins isolated from culture supernatant (Su). For Western blotting, proteins were electrophoretically transferred from the SDS-gel to a polyvinylidene difluoride (PVDF) membrane using a Mini Trans-Blot® Electrophoretic Transfer Cell (BioRad) following the manufacturer recommendations. XcpQ, ToxA and DsbA were detected by incubating the membranes with specific polyclonal antibodies diluted 1:5000, 1:5000, or 1:50000, respectively, with TBST buffer (0.1 M Tris-HCl, 0.1 M NaCl, pH 7.5, Tween 20 0.5% v/v), followed by an incubation with anti-rabbit IgG-horseradish peroxidase conjugate antibodies (Bio-Rad). The blots were developed with an ECL Western blotting detection kit (GE Healthcare). (PDF) [file pone.0069125.s005.pdf]

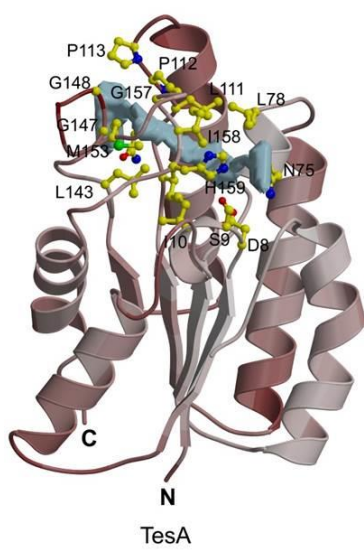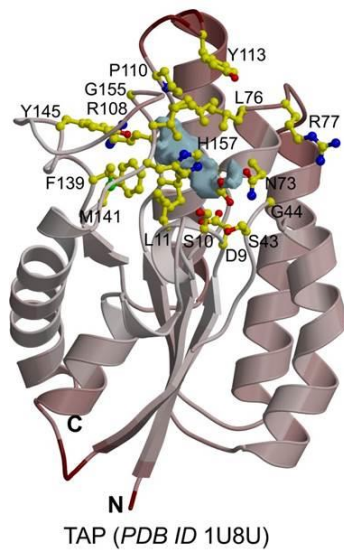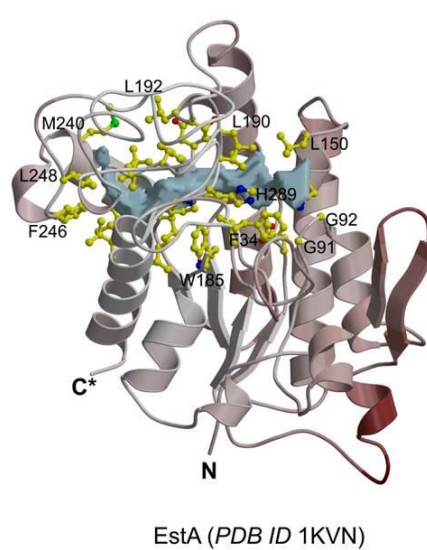

Supplement: Figure S3 — Residues involved in the formation of substrate binding cavities and channels in the GDSL family hydrolases TesA, Tap, and EstA calculated using program VOIDOO [43].. (PDF) [file pone.0069125.s006.pdf]

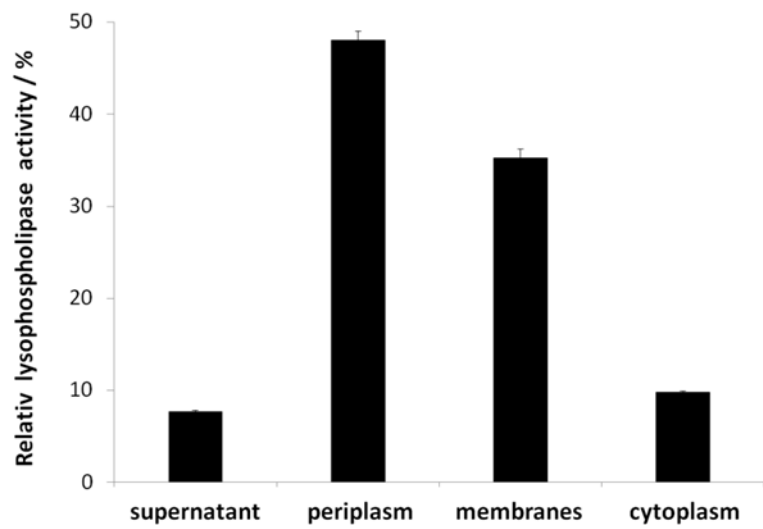

Supplement: Figure S5 — Lysophospholipase A activity was measured with C16-PGC as the substrate. A P. aeruginosa PA01 culture grown in LB medium at 37°C to stationary phase was used for cell fractionation. The assays were performed with 25 µL of fractions isolated from one ml of cell culture with OD580nm of 1. The relative activities were calculated by dividing the absolute activity of each fraction by the total lysophospholipase activity detected in cell extracts of P. aeruginosa PA01. (PDF) [file pone.0069125.s008.pdf]

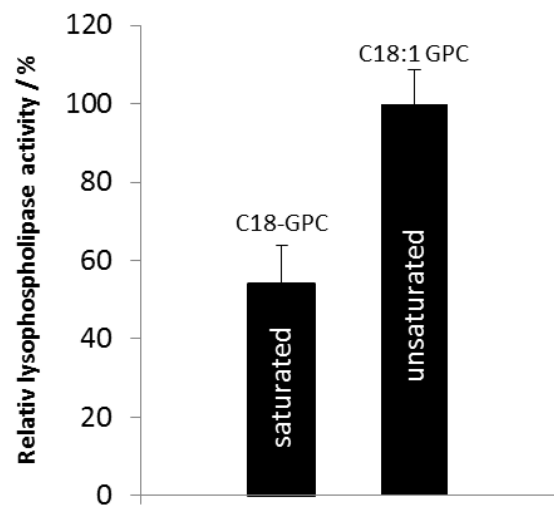

Supplement: Figure S6 — In the assay, 1 µg of purified TesA and 0.67 mM substrate (1-stearoyl-glycerophosphocholine C18-GPC, or 1-oleoyl-glycerophosphocholine, C18: 1-GPC) was used as described in Methods section. Activity of TesA with C18: 1-GPC was taken as 100%. (PDF) [file pone.0069125.s009.pdf]
