# Supplementary material for: Structural and Functional Characterisation of TesA - A Novel Lysophospholipase A from Pseudomonas aeruginosa
Source: PLoS One. 2013 Jul 18;8(7):e69125. doi: 10.1371/journal.pone.0069125 (PMC3715468; doi:10.1371/journal.pone.0069125)
Supplement: Figure S4 — Details of sequences are given in Table S3. Identical and similar amino acids are depicted on black and gray background. (PDF) [file pone.0069125.s007.pdf]

```
P. brassicacearum     MRVWFLSAGIALMCMAQNAAAGTVLIVGDSISAAFGLDTRQGWVSLLEQRLKAEGFDDKVVNASISGDTSAGGQARLEALLAAHKPDLVILELGGNDGLRGQPPTQLQCN 110
P. fluorescens        MRVWFLSAGIALMCMAQNAAAGTVLIVGDSISAGFGLDTRLGWVSLLEQRLKSEGFDDKVVNASISGDTSAGGQARLEALLAEHKPELVILELGGNDGLRGMPPTQLQCN 110
P. protegens          MRVWFLSAGIALMCMAQNAAAGTVLIVGDSISAAFGLDTRQGWVALLEKRLKDQGFNDRVVNASVSGDTSAGGQARLEALLAEHKPEVVILELGGNDGLRGQPPRQLQCN 110
P. chlororaphis       MRVWFLSAGIALMCMAQNAAAGTVLIVGDSISAAFGLDTRQGWVALLEQRLKSEGFDDEVVNASISGDTSAGGQARLEALLAEHKPELVILELGGNDGLRGQLPTQLQCN 110
P. synxantha          MRMWFLSAGIALMCMAQNAAAGTVLIVGDSISAGFGLDTRKGWVALLEQRLKQEGFDDKVVNASLSGDTSAGGLARLEAALAEHKPDVVVIELGGNDGLRGQPPAQLKCN 110
P. syringae           MRALFLSAGLGLLLLSQGALAGTVLIVGDSISAAFGLDTRVGWVSLLEQRLSKEGFDDKVVNASISGDTSAGGQARLEALLAEHKPELVILELGGNDGLRGQQPAQLQCN 110
P. viridiflava        MRAWFLSAGLGLLLLSQGAVAGTVLIVGDSISAAFGLDTRVGWVTLLEKRLKEQGFDDKVINASISGDTSAGGQARLEALLAEHKPELVILELGGNDGLRGQLPAQLQCN 110
P. entomophila        MRMWWLSAGIALCCLAQGAVAGTVLVVGDSISAGFGLDTREGWVELLQKRLKDEGFDDQVVNASISGDTSAGGRARLEALLVAHKPDLVVLELGGNDGLRGQPPQQLQCN 110
P. putida             MRVWWLSAGIALYCLAQSAAAGTLLVVGDSISAGFGLDSRQGWVSLLQARLRDEGFDEKVVNASISGDTSAGGQARLEALLAAHKPSLVVLELGGNDGLRGQSPAQLQCN 110
P. aeruginosa         MRALLLSGDIALVLLTQQAAAQTLLVVGDSISAALGLDTSQGWVALLQKRLADEGYDYRVVNASISGDTSAGGLARLEALLAEEKPALVVIELGGNDGLRGMAPAQLQCN 110
P. mendocina          MRAWWLSGAIALLFWAQGAVAGTLLVVGDSISAAFGLDSRQGWVALLEKRLREQGFDHAVVNASISGDTSAGGAARLEALLAEHRPSLVIIELGGNDGLRGQPPAQLQCN 110
P. pseudoalcaligenes  MRAWLLGGDLSLLLLIAQEAVAGTVLVVGDSISAALGLESSQGWVSLLEKRLVEKGYDKQVVNASISGDTSAGGLSRLEALLAEHKPELVIIELGGNDGLRGQPPAQLQCN 110
P. fulva              MRAIVKGAMLGLLLIGQMAQAGTVLVVGDSISAAFGMDTQQGWVHLLDERMREEGFEHQIVNASISGDTSAGGAARLEALLTEHKPEVVIVELGGNDGLRGLPPSQLQCN 110
P. stutzeri           MRRWLKDGAIALLCWTQGALAGTVLVVGDSISAAFGLETSQGWVHLLQERLVEGDESWRVVNASISGDTTAGGLARLDPLLEEHTPEVVILELGGNDGLRGQSPVQLKCN 110
                      **     . *.*    *  *  * *:*:*******.:*:::  *** **: *:    . .   ::***:****:***  :**  .  *    .  * :*::**********   *  **:** 70

P. brassicacearum     IAAMIDSARASGAQVILLGMQLPENYGRRYTDAFARVYSTIAEEKKVPLVEFFLKDVGGIPTMMQGDGLHESVAAAQGKLIENVWETLKPLL 201
P. fluorescens        IASMIDSSRQSGAKVILLGMQLPENYGKRYTDAFAEVYGKIAEEKKIPLVEFFLDGVGGHPDIMQADGLHEAAGAQGKLIENVWETLKPLL 201
P. protegens          IASMIDSSQAQGAKVILLGMQLPENYGVRYTQAFSQVYSEIASEKKVALVEFFLDGVGGHPEIMQADGLHEAAAAQGKLIENVWETLKPLL 201
P. chlororaphis       IASMIDSSKASGAKVILLGMQLPENYGVRYTQAFAEVYSKIANEKNVALVEFFLEGVGGNPEIMQADGIHEAVAAQGKLIENVWETLKPLL 201
P. synxantha          IASMIDQSKAGGAKVILLGMQLPENYGPRYTTAFAEVYGVIAKEKNVPLVEFFLEGVGGHPEIMQADQLHEAVAAQGKLIENVWETLKPLL 201
P. syringae           LSSMIDRSQAAGAKVILLGMRIPENYGPRYTKAFEEVYSNIAEEKKIPFVEFFLEGVGGVPEIMQADGLHEAAAAQGKLIENVWESLKPLL 201
P. viridiflava        LSSMIDSSRAAGAKVILLGMRIPENYGPRYTKAFEEVYSNIAEEKKVPFVEFFLEGVGGVPQIMQADGLHEAAAAQGKLIENVWETLKPLL 201
P. entomophila        IALMIDFARQAGARVVLLGMRLPENYGVRYTTAFAQVYEQIASDKQVPLVEFFLEGVGGVPAMMQADGIHEAPVAQQRLIENAWETIKPLL 201
P. putida             IASMIERSRQAGAKVILLGMRLPENYGVRYTTAFAKVYEQIAADKQVPLVEFFLEGVGGVPEIMQADGIHEAQGAQQRLIENAWEAIKPLL 201
P. aeruginosa         IASMAQKARAEGAKVILLGIQLPENYGPRYIEAFSRVYGAVAAQEKTALVEFFLEGVGGVQGMMQADGIHEALAAQPRLIENVWETLKPLL 201
P. mendocina          IASMIEQSQAAGARVILLGMRLPENYGARYTSAFAQVFADIAEQKQVPLVEFFLEGVGGVPAMMQDDGIHEALAAQEKLIENVWESLQPLL 201
P. pseudoalcaligenes  IAGMIDSSRAQGAAVILLGMRLPENYGARYTSAFAKVYSDIAEQKQVPLVEFFLEGVGGVPEIMQPDGIHPQANAQPRLIENVWETLEPLL 201
P. fulva              IASMIDASRNAGAKVILLGMRIPENYGERYTTAFAKVYGDIAADKKVPLVEFILDGVGGVASIMQDDGVHEAVAAQPQLIENVWETLKPLL 201
P. stutzeri           IADMIDRSREAGAEVILLGMRMPENLGQRYTRAFADAFDSIAQEKSVAYVEFILEGVGGVAGMMQADGIHETAEAQSQLIETVWEALEPLL 201
                      *: *  :  ::    **  *:***:::*** * **   **    .:   :* ::. .  ***:*..***     :** * :**    **  :***..**:::*** 128
```
